# Knowledge and perception of mHealth medication adherence applications among pharmacists and pharmacy students in Jazan, Kingdom of Saudi Arabia

**DOI:** 10.1371/journal.pone.0308187

**Published:** 2024-08-30

**Authors:** Wajiha Rehman, Hemalatha Thanganadar, Sumaira Idrees, Asim Mehmood, Fahad Khan Azeez, Hanan Abdullah Almaimani, Pushp Lata Rajpoot, Mohammed Mustapha

**Affiliations:** 1 Department of Health Informatics, College of Public Health and Tropical Medicine, Jazan University, Jazan, Kingdom of Saudi Arabia; 2 Department of Health Education and Promotion, College of Public Health and Tropical Medicine, Jazan University, Jazan, Kingdom of Saudi Arabia; 3 QU Health, Qatar University, Doha, Qatar; Lahore Medical and Dental College, PAKISTAN

## Abstract

The advances in digital health, including mobile healthcare (mHealth) medication adherence applications (MApps), have been demonstrated to support medication adherence and improve health outcomes. This study aims to evaluate the knowledge and perception of the MApps among pharmacists and pharmacy students. An online cross-sectional survey was conducted among 223 pharmacists and pharmacy students in the Jazan region of Saudi Arabia between 1st and 30th April 2023. The survey collected information about the participants’ socio-demographics, knowledge, and perception of the MApps. Among the 223 participants included in the study, 105 (47.1%) were pharmacists and 118 (52.9%) were pharmacy students. Most participants were females (72.6%) and aged 18–30 (70.4%). About half of the participants had poor knowledge of the MApps [pharmacists (48.0%) and students (42.0%)] and mainly encountered *Medisafe* (18.1%) or *Pills* (17.0%) MApps, respectively. Pharmacy students showed significantly higher knowledge of MApps (*p* = 0.048), especially the *Pills* (*p* = 0.022) than pharmacists. However, the pharmacists had significantly higher knowledge of *MyMeds* (*p* = 0.001) than pharmacy students. Most participants had a positive perception of the usefulness of the MApps (pharmacists, 79.0%; students 80.0%). Notably, over 85% of the participants expressed willingness to know and provide guidance on MApps, with over 50% willing to recommend it to the patients. There was no significant difference in perception between the pharmacists and pharmacy students (*p*>0.05). In conclusion, the study demonstrates limited knowledge with a positive perception of mHealth medication adherence applications among pharmacists and pharmacy students. Integrating digital adherence tools like the MApps into pharmacy training could significantly improve professional practice mHealth competencies, and optimize healthcare delivery and patient outcomes.

## Introduction

Chronic diseases are the leading causes of death, disabilities, and healthcare costs globally [1, 2]. Chronic diseases can be effectively managed through long-term medication and lifestyle support, with medication adherence playing a pivotal role in improving health outcomes [3–5]. Medication non-adherence remains an issue of public health concern, especially in those people living with chronic disease [6, 7]. Moreover, the prevalence of chronic diseases and the number of individuals requiring long-term medication therapy are on the rise with the global increasing life expectancy [7]. Consequently, individuals who do not adhere to their medications are at risk of poor treatment outcomes [8]. Recognizing the critical nature of adherence, the World Health Organization (WHO) has identified medication non-adherence as a priority healthcare problem that is preventable [9].

Medication adherence is a complex behavior influenced by multiple factors spanning patient, provider, and health system-related domains [10]. Three distinct methods are commonly employed to assess medication adherence: patient self-reports, medication refill data, and electronic monitoring devices [11–13]. While self-reporting is susceptible to recall and reporting bias, refill records merely indicate the quantity of prescribed medication without confirming actual consumption. Electronic devices are often considered the gold standard for adherence assessment due to their ability to record dosing events and timing [12]. Measuring adherence through prescription drug refills can be a cost-effective and practical approach if a standardized medication regimen is in place [14]. This indirect method allows for assessing adherence rates without the patient’s knowledge, enhancing the accuracy of estimates [15]. However, it does have limitations, such as the inability to classify irregular refill patterns and the assumption that a filled prescription equates to medication consumption. Electronic monitoring devices such as mobile medication adherence apps could be the best cost-effective alternative.

Mobile healthcare (mHealth) medication adherence applications are new advancements in digital technology to improve medication adherence and health outcomes [16]. The mHealth represents a versatile and interactive solution that bridges the gap between SMS reminders and electronic pillboxes, offering a more affordable and practical alternative [16, 17]. The accessibility, affordability, and diversity of medication adherence apps make them a compelling choice for managing chronic diseases. These mobile apps offer a range of features, including medication reminders, which can significantly assist patients in adhering to their prescribed regimens and minimizing medication errors [16]. However, the mHealth medication adherence apps approach would require adequate knowledge and competence of the healthcare professionals, especially the pharmacy professionals, which has not been explored.

Over the last few years, the number of smartphone users in Saudi Arabia has risen steadily, exceeding 30 million [18]. As a result, the market has witnessed a surge in mHealth apps. Mobile technology’s rapid evolution has made it a ubiquitous presence in healthcare, and leveraging mHealth apps can enhance patient medication compliance, optimize outcomes, and reduce medication wastage [19]. Although there are many mobile apps for medication adherence, there is limited evidence of their understanding among healthcare professionals, especially those in pharmacy. One review study on cardiovascular disease suggested that mobile apps effectively improved medication adherence [20]. However, the main target of the study was patients’ clinical outcomes rather than medication adherence. Globally, the most common medication adherence mobile apps are identified as *Medisafe*, *MyMeds*, and *Pills* [21]. A study found that *Medisafe* improved awareness of medication adherence and reduced self-reported barriers [22]. Understanding the perspective of health professionals, especially among pharmacy stakeholders, could support patients’ optimal utilization of the apps to improve treatment outcomes.

Pharmacists play a crucial role in improving medication adherence [23]. Pharmacists’ most significant role is to support the patients in identifying techniques to improve medication adherence and, consequently, health outcomes [24]. Despite the critical role of medication adherence mobile apps in improving patients’ outcomes, there is a lack of evidence on the level of knowledge of pharmacists and pharmacy students regarding mHealth apps. Therefore, to improve medication adherence among end-users through mHealth-based apps, it is imperative to understand the perspectives of the key stakeholders in the pharmacy profession. Therefore, this study aimed to assess the knowledge and perception of mHealth medication adherence applications among pharmacists and pharmacy students in Jazan, Kingdom of Saudi Arabia.

## Materials and methods

### Study design

A cross-sectional study was conducted among pharmacists and pharmacy students in the Jazan region of the Kingdom of Saudi Arabia (KSA), between 1st and 30th April 2023. Data were collected using a structured self-administered online survey developed using Google Forms, that assessed the knowledge and perceptions of mHealth medication adherence applications (MApps). The study adhered to the guidelines outlined in the Standard Reporting System for Observational Studies (STROBE) [25], and as utilized elsewhere [26, 27]. The STROBE checklist was incorporated into our study reporting to ensure transparency and methodological rigor.

### Study population and setting

The study encompassed a diverse population of adult pharmacists aged 18 years and above who are employed in various sectors, such as public and private hospitals, as well as community pharmacies and NGOs within the Jazan region of the KSA. Additionally, adult pharmacy students aged 18 years and above who are enrolled at the Pharmacy College of Jazan University from all academic levels were included in this study. The study excluded individuals who declined to participate and those outside the pharmacy profession. Our study attempted to cover the general scope of the pharmacy profession from training (pharmacy students) to practice (pharmacists).

### Sample size and sampling method

The minimum sample size for the study was estimated based on the prevalence and proportion formula using OpenEpi: Open Source Epidemiologic Statistics for Public Health online software version 3.01 [28]. Population size of the targeted participants (N): 1,000; Hypothesized (%) frequency of outcome in the population (p): 50% +/-5; Confidence limits as % of 100 (absolute +/%) (d): 5%; Design effect (DEFF): 1; Z is a constant = 1.96 for 95% confidence interval (CI). Therefore, the minimum required sample size for the study was estimated to be 213. The study participants were recruited through convenience, and snowball sampling methods were employed to improve the representation of the responses. These sampling approaches were chosen for their practicality, ease of implementation, and cost-effectiveness, aligning with the study’s goals of efficiently reaching a diverse online audience.

### Data collection

Data collection was facilitated through an adapted structured survey tool designed to assess knowledge and perception of MApps [29, 30]. The survey collected information related to the participants’ socio-demographics, knowledge, and perceptions toward the MApps. The drafted survey was shared with three experts in pharmacy who confirmed all items to be relevant and clear. A pilot study was conducted among fifteen pharmacists and pharmacy students (a total of thirty participants) to verify the comprehensibility of the questionnaire. All questions were deemed unambiguous. The internal consistency reliability of the survey tool was checked and was found to be reliable with Cronbach alpha coefficients of 0.82 (knowledge domain) and 0.79 (perception domain). The responses from the pilot were excluded from the main study.

The final validated survey was disseminated to potential pharmacists within the study frame (including university, hospitals and community pharmacies located within Jazan, KSA). Additionally, the questionnaire was distributed across pharmacy students enrolled at the College of Pharmacy, Jazan University, KSA. The online survey was distributed via popular social media platforms, including Facebook, Telegram, and Twitter, and via emails to reach diverse and representative pharmacists and pharmacy students.

### Data analysis

Data analysis was performed using IBM SPSS Statistics version 28.0. To ensure data quality, the data underwent thorough cleaning, consistency checks, and completeness verification. Descriptive analysis techniques were applied to summarize and present the collected data, providing insights into participants’ knowledge and perceptions regarding smartphone-based medication adherence applications. This approach facilitated the comprehensive characterization of the study population and the elucidation of key findings in line with the STROBE reporting guidelines [25].

### Ethical statement

All procedures performed in studies involving human participants were in accordance with the ethical standards of the Institutional Review Board (IRB) and the 1964 Helsinki Declaration. The study was granted an exemption of ethical approval by the Research and Human Ethics Standing Committee of the College of Public Health and Tropical Medicine, Jazan University, Jazan, KSA, with the reference JAZANU/CPHTM/HRESC/01/03/23. The committee has determined that the survey study poses no undue risk to participants and is in line with ethical standards for research involving human subjects. The committee also acknowledges the voluntary nature of participation, the assurance of confidentiality and anonymity, and the relevance of the study to the local context. All participants involved in this study provided online written informed consent prior to their participation and as approved by the committee. Participants were presented with detailed information about the study’s objectives, procedures, and the potential risks and benefits of participation. The participants were then prompted to select accept or reject options for participating in the study.

## Results

### Socio-demographic characteristics of study participants

Table 1 summarizes the socio-demographic characteristics of the included study participants. The final analysis included two hundred and twenty-three (223) participants. The response rate for the survey was 75%, calculated based on the number of completed surveys received (223) out of approximately 300 who clicked the link invitations sent out. Participants were mostly females 162 (72.6%), aged 18–30 years 199 (89.2%), and of Saudi origin 208 (93.3%). The participants were distributed into pharmacists 105 (47.1%) and pharmacy students 118 (52.9%). Among the pharmacists, the majority had 1–3 years of experience 63 (63.6%), working in the hospital 43 (43.4%), community 29 (29.3%), or NGOs 27 (27.3%).

**Table 1:** Socio-demographic characteristics of the study participants.

Parameters	Frequency (%), N = 223
**Gender**	
Female	162 (72.6)
Male	61 (27.4)
**Age (years)**	
18–30	199 (89.2)
31–45	18 (8.1)
46–65	5 (2.2)
Above 65	1 (0.4)
**Marital status**	
Single	157 (70.4)
Married	61 (27.4)
Divorced	5 (2.2)
**Nationality**	
Saudi	208 (93.3)
Non-Saudi	15 (6.7)
**Pharmacy qualification**	
Pharmacists	105 (47.1)
Pharmacy Students	118 (52.9)
**Years of experience (pharmacists)**	
0–3 years	63 (63.6)
4–6 years	21 (21.2)
7–10 years	7 (7.1)
11 years or above	8 (8.1)
**Area of practice (pharmacists)**	
Hospital	43 (43.4)
Community	29 (29.3)
NGOs	27 (27.3)

Keys: NGO = Non-governmental organizations

### Knowledge of mHealth medication adherence applications

Regarding the knowledge about mHealth medication adherence applications, about half of the pharmacists 50 (48.0%) and pharmacy students 50 (42.0%) don’t know the MApps. The type of MApps the pharmacists and students encountered were *Medisafe* 19 (18.1%) and *Pills* 20 (17.0%), respectively. There was a statistically significant difference in the knowledge of MApps between pharmacists and pharmacy students (*p* = 0.048), with students showing slightly more knowledge than pharmacists. In addition, pharmacists and students had a statistically significant difference in using the *MyMeds* (*p* = 0.001) and Pills (*p* = 0.022) MApps, with the pharmacists demonstrating more knowledge of the *MyMeds* and pharmacy students having more knowledge of the *Pills*. Table 2.

**Table 2:** Knowledge of mHealth medication adherence applications.

Parameters	Pharmacists, n (%) (N = 105)	Pharmacy Students, n (%) (N = 118)	*p*-values
**MApps knowledge**			
Yes	55 (52.0)	68 (58.0)	0.048
No	50 (48.0)	50 (42.0)	
**MApps encountered**			
*Medisafe*	19 (18.1)	13 (11.0)	0.207
*MyMeds*	9 (8.6)	1 (0.9)	0.001
*Pills*	7 (6.8)	20 (17.0)	0.022

Keys: MApps = mHealth medication adherence applications

### Perception of mHealth medication adherence applications

Most of the participants agreed that MApps are useful [pharmacists 83 (79.0%) and pharmacy students 94 (80.0%)]. They also agreed that the MApps are useful to all types of patients [pharmacists 79 (75.0%) and students 89 (75.0%)]. The participants mostly agreed that the scenario of using MApps was a reminder to take medicines [pharmacists 45 (43.0%) and students 58 (49.0%)]. There was no statistically significant difference in the perception of MApps between pharmacists and pharmacy students (*p*>0.05) Table 3.

**Table 3:** Perception of mHealth medication adherence applications.

MApps Parameters	Pharmacists, n (%) (N = 105)	Pharmacy Students, n (%) (N = 118)	*p*-values
**Overall usefulness**			
Yes	83 (79.0)	94 (80.0)	0.873
No	22 (21.0)	24 (20.0)	
**Usefulness to patients**			
All patients	79 (75.0)	89 (75.0)	1.000
Chronic disease	13 (12.0)	11 (9.0)	0.829
Communicable disease	1 (1.0)	4 (3.0)	0.366
Busy patients	5 (5.0)	5 (4.0)	0.837
Short memory	7 (7.0)	11 (9.0)	0.641
**Approach for use**			
Application	31 (29.0)	37 (31.0)	0.771
Disease nature	14 (13.0)	6 (5.0)	0.159
Reminders	45 (43.0)	58 (49.0)	0.203
Counselling	14 (13.0)	13 (11.0)	0.831
Mixed (All)	(0)	2 (2.0)	-

Keys: MApps = mHealth medication adherence applications

### Willingness to support mHealth medication adherence applications

The majority of the participants were willing to know more about the MApps [pharmacists 91 (87.0%); students 104 (88.0)], and willing to guide the patients on the use of MApps [pharmacists 91 (87.0%); students 106 (90.0%)], but with just about half willing to recommend the use of MApps to the patient, relatives, or friends [pharmacists 58 (55.0%); students 68 (58.0%)] There was no statistically significant difference in the willingness to support MApps between pharmacists and pharmacy students (*p*>0.05) Table 4.

**Table 4:** Willingness to support mHealth medication adherence applications.

Willingness Parameters	Pharmacists, n (%) (N = 105)	Pharmacy Students, n (%) (N = 118)	*p*-values
**Know MApps**			
Yes	91 (87.0)	104 (88.0)	0.786
No	13 (12.0)	11 (9.0)	
Not sure	1 (1.0)	3 (3.0)	
**Guide on MApps**			
Yes	91 (87.0)	106 (90.0)	0.460
No	12 (11.0)	7 (6.0)	
Not sure	2 (2.0)	5 (4.0)	
**Recommend MApps**			
Yes	58 (55.0)	68 (58.0)	0.757
No	44 (42.0)	41 (35.0)	
Not sure	3 (3.0)	8 (7.0)	

Keys: MApps = mHealth medication adherence applications

## Discussion

This study was the first to investigate the knowledge and perception of mHealth medication adherence applications among pharmacists and pharmacy students in Jazan, Kingdom of Saudi Arabia. The current study found a significant proportion of the participants with limited knowledge but a positive perception of the MApps. Addressing this gap through expanded awareness programs during pharmacy training and professional practice could optimize the end-user application of the MApps in improving medication adherence and overall treatment outcomes. Moreover, the participants’ willingness to recommend the mobile apps emphasizes the potential to enhance their professional competence and elevate their expertise and confidence in chronic disease management. The significance of the study lies in its focus on a population critical to the healthcare system, as pharmacists play a leading role in medication management and patient education. Integrating digital health tools, such as MApps, in pharmacy training and practice will advance the roles and improve patient outcomes.

The study revealed that nearly half of the participants, pharmacists and students, exhibited limited knowledge of MApps. This features a critical gap in the awareness of mHealth tools within the pharmacy profession. Using mHealth tools represents an advanced approach to supporting medication adherence, demonstrating promising outcomes in some studies. One study reported that very few participants had used a medication adherence app. Despite the limited awareness, there was a noteworthy level of interest, with more than half of the respondents expressing a strong interest in downloading the apps [31]. The lack of awareness could also be attributed to satisfaction with traditional adherence methods and the possible missing link from the healthcare providers, especially in the pharmacy, who take leading roles in medication adherence counseling.

The participants reported very low encounters with the most commonly available MApps, including *Medisafe*, *MyMeds*, and *Pills*. These apps were developed to address non-adherence issues [32]. They provide alerts to patients when it is time to take medication. *Medisafe* allows patients to generate weekly medication adherence reports and monitor biometric measurements. The app designates a *"Medfriend"* of their choosing, who is granted access to a patient’s medication-taking history, receives alerts when doses are missed, and can provide peer support. A study found that *Medisafe* use in diabetes management was feasible and acceptable, improved awareness of medication adherence, and reduced self-reported barriers to medication adherence [22]. This suggests that the mHealth Apps market holds a significant influence over individuals’ choices when it comes to medication adherence Apps. Understanding the factors contributing to the popularity of these specific apps can be valuable for developers and healthcare providers looking to optimize their impact. Providing in-depth training on the functionalities and advantages of the widely used MApps can empower the pharmacists and other healthcare professionals to recommend and guide patients effectively.

While mHealth apps (*Medisafe*, *MyMeds*, and *Pills*) hold significant promise as reminders for dosing regimens, they often fail to address educational barriers and health literacy among users. These challenges can be effectively tackled through service-driven support mechanisms within the community pharmacy setting, ensuring patients understand their prescribed medications and associated dosing schedules clearly. An exemplary illustration of an advanced system addressing medication adherence is *SmartTrack*, an innovative device designed to enhance adherence to inhaler devices. Foster and colleagues [33] revealed that, after six months, *SmartTrack* users exhibited significantly higher levels of medication adherence.

Despite the limited knowledge, the study found a positive perception of the usefulness of the MApps and willingness to guide and recommend the app among pharmacists and pharmacy students. This positive attitude is crucial for successfully integrating digital health tools into pharmacy training and practice. It suggests that once educated about MApps, pharmacists, and students will likely embrace these technologies as valuable resources for improving medication adherence competence. Comparable to other regional studies in the KSA, one study reported that most pharmacy students showed positive perceptions regarding telehealth apps (a more generic mHealth tool) [34], and another study reported positive perception among healthcare professionals and medical students on the use of technology to enhance adherence [35]. The willingness expressed by over 85% of the participants to learn about and guide patients on MApps signifies a potential readiness among pharmacists and students to incorporate MApps into their professional activities. Harnessing this enthusiasm through structured training programs can lead to a more proficient and informed healthcare workforce.

The study has several limitations that should be considered when interpreting its findings. Firstly, the composition of the sample may introduce bias. Most participants in this study were females aged 18–30 from the Jazan region of Saudi Arabia. While this demographic profile may be representative of the study’s specific geographical context, it may not accurately reflect the broader diversity of pharmacists and pharmacy students in the entire country. Consequently, the generalizability of the study’s findings could be limited. Secondly, the study’s cross-sectional design, while valuable for capturing a snapshot of participants’ knowledge and attitudes, does not provide insights into how these factors may change over time. Causality cannot be inferred from cross-sectional data and important temporal trends or variations in awareness and attitudes may be missed. Future research employing longitudinal designs could offer a more comprehensive understanding of the evolution of knowledge and attitudes in this context. Such studies can offer valuable insights into the effectiveness of educational initiatives and the sustained benefits of digital health interventions in improving medication adherence and, ultimately, patient health. Thirdly, the study relies on self-reported data, including participants’ knowledge levels, app usage patterns, and perception levels. This reliance on self-reporting introduces potential sources of bias. For example, participants may not accurately recall their app usage or may provide responses that they believe are socially desirable. Future research could consider supplementing self-report data with objective measures of app usage and knowledge to mitigate such biases. Lastly, the study’s geographic scope is limited to the Jazan region of Saudi Arabia. While this regional focus aligns with the study’s specific context, it may not capture variations in healthcare contexts, digital health infrastructure, and digital health adoption rates in other regions or countries. A broader geographic scope can provide insights into potential regional variations and inform tailored interventions.

Despite these limitations, the study offers several notable strengths that enhance its value and contribution to the field. Firstly, the study addresses a highly relevant issue in contemporary healthcare—the challenge of medication adherence among individuals with chronic diseases. In doing so, it explores the potential of technology, specifically medication adherence apps, to address this challenge. Given the increasing reliance on digital health solutions in today’s technologically driven world, this research topic is relevant and important. Secondly, the study benefits from a diverse sample, including pharmacists and pharmacy students. This diversity allows for a broader perspective on the awareness and perceptions of medication adherence apps among individuals at different stages of their healthcare careers. Understanding how knowledge and attitudes vary between these two groups can provide valuable insights for educators and healthcare professionals. Thirdly, the study employs a rigorous quantitative data collection and analysis approach. Using descriptive and inferential statistics adds rigor to the study’s findings, providing quantifiable insights into the knowledge and attitudes of the participants. This quantitative approach enables a structured and systematic examination of the research questions. Furthermore, the study’s findings have practical implications for healthcare professionals and educators. It emphasizes the importance of educating pharmacy students and practicing pharmacists about digital health tools and their potential role in patient care. As digital health continues to play an increasingly prominent role in healthcare, this emphasis on education and training is crucial for ensuring that healthcare providers are well-prepared to navigate this evolving landscape.

### Conclusions

Our study demonstrated a limited level of knowledge with a positive perception of mHealth medication adherence applications (MApps) among pharmacists and pharmacy students in Jazan, Kingdom of Saudi Arabia. The MApps offer an innovative means to improve patient adherence to medication, thus optimizing treatment outcomes. There is substantial room for improvement in promoting awareness of the apps among healthcare stakeholders, including patients and healthcare professionals, through educational programs, workshops, or integrated courses. Collaborative efforts with healthcare institutions can be pivotal in promoting mHealth and fostering a culture of improved medication adherence. Furthermore, collaboration with the app developers should be encouraged to ensure continuous MApps user feedback to enhance features and overall user satisfaction.

## References

[1] Harris RE. Epidemiology of chronic disease: global perspectives: Jones & Bartlett Learning; 2019.

[2] Anderson E, Durstine JL. Physical activity, exercise, and chronic diseases: A brief review. Sports Medicine and Health Science. 2019;1(1):3–10. doi: 10.1016/j.smhs.2019.08.006 35782456 PMC9219321

[3] Subramanian M, Wojtusciszyn A, Favre L, Boughorbel S, Shan J, Letaief KB, et al. Precision medicine in the era of artificial intelligence: implications in chronic disease management. Journal of translational medicine. 2020;18(1):1–12. 33298113 10.1186/s12967-020-02658-5 PMC7725219

[4] Chan SW-C. Chronic disease management, self-efficacy and quality of life. Journal of Nursing Research. 2021;29(1):e129. doi: 10.1097/JNR.0000000000000422 33427791 PMC7808345

[5] Wilson TE, Hennessy EA, Falzon L, Boyd R, Kronish IM, Birk JL. Effectiveness of interventions targeting self-regulation to improve adherence to chronic disease medications: A meta-review of meta-analyses. Health Psychology Review. 2020;14(1):66–85. doi: 10.1080/17437199.2019.1706615 31856664 PMC7254887

[6] Cheen MHH, Tan YZ, Oh LF, Wee HL, Thumboo J. Prevalence of and factors associated with primary medication non‐adherence in chronic disease: a systematic review and meta‐analysis. International journal of clinical practice. 2019;73(6):e13350. doi: 10.1111/ijcp.13350 30941854

[7] Foley L, Larkin J, Lombard-Vance R, Murphy AW, Hynes L, Galvin E, et al. Prevalence and predictors of medication non-adherence among people living with multimorbidity: a systematic review and meta-analysis. BMJ open. 2021;11(9):e044987. doi: 10.1136/bmjopen-2020-044987 34475141 PMC8413882

[8] Walsh CA, Cahir C, Tecklenborg S, Byrne C, Culbertson MA, Bennett KE. The association between medication non‐adherence and adverse health outcomes in ageing populations: a systematic review and meta‐analysis. British journal of clinical pharmacology. 2019;85(11):2464–78. doi: 10.1111/bcp.14075 31486099 PMC6848955

[9] Organization WH. World patient safety day goals 2020–21: health worker safety: a priority for patient safety. World Health Organization, 2020.

[10] Basnet R, Bhandari R, Gautam S, Lama N, Shreejana K, Bhandari TR. Does Patient Counselling Correspond to Medication Adherence in Chronic Diseases?-A Scoping Review. Journal of Karnali Academy of Health Sciences. 2021;4(3):1–11.

[11] Anghel LA, Farcas AM, Oprean RN. An overview of the common methods used to measure treatment adherence. Medicine and pharmacy reports. 2019;92(2):117. doi: 10.15386/mpr-1201 31086837 PMC6510353

[12] Shah KK, Touchette DR, Marrs JC. Research and scholarly methods: Measuring medication adherence. Journal of the American College of Clinical Pharmacy. 2023;6(4):416–26.

[13] Mohammed MM. ORIGINAL RESEARCH Assessment of Information Communication Technology and eHealth Use Amongst Healthcare Providers in Selected Primary Health Centers in Kano, Nigeria Ahmed MA1, 2, Mohammed M3, 4, Haruna U5, 6, Mustapha S7, 8, Wada Y9, 10, Ladan SM11, Muhammad S12, Zaji FM13, Ahmed MH8 and Sha’aban A3, 4. Journal of Basic and Social Pharmacy Research. 2022;2(5):1–10.

[14] Jose J, Bond C. Medication Adherence: still a problem. Oxford University Press UK; 2021. p. 93–5. 10.1093/ijpp/riaa019 33729522

[15] Galozy A, Nowaczyk S, Sant’Anna A, Ohlsson M, Lingman M. Pitfalls of medication adherence approximation through EHR and pharmacy records: definitions, data and computation. International Journal of Medical Informatics. 2020;136:104092. doi: 10.1016/j.ijmedinf.2020.104092 32062562

[16] Pérez-Jover V, Sala-González M, Guilabert M, Mira JJ. Mobile apps for increasing treatment adherence: systematic review. Journal of medical Internet research. 2019;21(6):e12505. doi: 10.2196/12505 31215517 PMC6604503

[17] Muro-Culebras A, Escriche-Escuder A, Martin-Martin J, Roldán-Jiménez C, De-Torres I, Ruiz-Muñoz M, et al. Tools for evaluating the content, efficacy, and usability of mobile health apps according to the consensus-based standards for the selection of health measurement instruments: systematic review. JMIR mHealth and uHealth. 2021;9(12):e15433. doi: 10.2196/15433 34855618 PMC8686474

[18] Aldhaban F, Daim T, Harmon R, Basoglu N. Technology adoption in emerging regions: Case of the smartphone in Saudi Arabia. International Journal of Innovation and Technology Management. 2020;17(01):2050003.

[19] Bond Z, Scanlon T, Judah G, editors. Systematic review of RCTs assessing the effectiveness of mHealth interventions to improve statin medication adherence: using the behaviour-change technique taxonomy to identify the techniques that improve adherence. Healthcare; 2021: MDPI. doi: 10.3390/healthcare9101282 34682962 PMC8535703

[20] Mohammadi R, Ayatolahi Tafti M, Hoveidamanesh S, Ghanavati R, Pournik O. Reflection on mobile applications for blood pressure management: a systematic review on potential effects and initiatives. Building Continents of Knowledge in Oceans of Data: The Future of Co-Created eHealth. 2018:306–10. 29677972

[21] Varshney U, Singh N. An analytical model to evaluate reminders for medication adherence. International Journal of Medical Informatics. 2020;136:104091. doi: 10.1016/j.ijmedinf.2020.104091 32036321

[22] Huang Z, Tan E, Lum E, Sloot P, Boehm BO, Car J. A smartphone app to improve medication adherence in patients with type 2 diabetes in Asia: feasibility randomized controlled trial. JMIR mHealth and uHealth. 2019;7(9):e14914. doi: 10.2196/14914 31516127 PMC6746066

[23] Spanakis M, Sfakianakis S, Kallergis G, Spanakis EG, Sakkalis V. PharmActa: Personalized pharmaceutical care eHealth platform for patients and pharmacists. Journal of Biomedical Informatics. 2019;100:103336. doi: 10.1016/j.jbi.2019.103336 31689550

[24] Hassan Y, Al-Temimi AA, Ramli R, Saad MFM, Aziz NA. Difficulties facing the patients and extra burden for healthcare providers by non-adherence to the medications-a comprehensive review. Indian Journal of Pharmaceutical Education and Research. 2019;53(4s):s487–s99.

[25] Vandenbroucke JP, Elm Ev, Altman DG, Gøtzsche PC, Mulrow CD, Pocock SJ, et al. Strengthening the Reporting of Observational Studies in Epidemiology (STROBE): explanation and elaboration. Annals of internal medicine. 2007;147(8):W-163-W-94. 10.7326/0003-4819-147-8-200710160-00010-w1 17938389

[26] Mohammed M, Kumar N, Zawiah M, Al-Ashwal FY, Bala AA, Lawal BK, et al. Psychometric Properties and Assessment of Knowledge, Attitude, and Practice towards ChatGPT in Pharmacy Practice and Education: A Study Protocol. Journal of racial and ethnic health disparities. 2023:1–10. doi: 10.1007/s40615-023-01696-1 37428357

[27] Mustapha M, Lawal BK, Sha’aban A, Jatau AI, Wada AS, Bala AA, et al. Factors associated with acceptance of COVID-19 vaccine among University health sciences students in Northwest Nigeria. PloS one. 2021;16(11):e0260672. doi: 10.1371/journal.pone.0260672 34843594 PMC8629299

[28] Dean A. OpenEpi: open source epidemiologic statistics for public health, version 2.3. 1. http://wwwopenepicom. 2010.

[29] Davies MJ, Kotadia A, Mughal H, Hannan A, Alqarni H. The attitudes of pharmacists, students and the general public on mHealth applications for medication adherence. Pharmacy practice. 2015;13(4). doi: 10.18549/PharmPract.2015.04.644 26759619 PMC4696122

[30] Ahmed I, Ahmad NS, Ali S, Ali S, George A, Saleem Danish H, et al. Medication Adherence Apps: Review and Content Analysis. JMIR Mhealth Uhealth. 2018;6(3):e62. doi: 10.2196/mhealth.6432 29549075 PMC5878368

[31] Abdullah NZL, Halim SA, Hamidi FW. Patient’s Perception on the Features of Interest and Barriers of Smartphone Medication Adherence Applications. PHARMACY RESEARCH REPORTS. 2020:29.

[32] Morawski K, Ghazinouri R, Krumme A, McDonough J, Durfee E, Oley L, et al. Rationale and design of the medication adherence improvement support app for engagement—blood pressure (*Medisafe*-BP) trial. American heart journal. 2017;186:40–7. 28454831 10.1016/j.ahj.2016.11.007

[33] Foster JM, Usherwood T, Smith L, Sawyer SM, Xuan W, Rand CS, et al. Inhaler reminders improve adherence with controller treatment in primary care patients with asthma. Journal of Allergy and Clinical Immunology. 2014;134(6):1260–8. e3. doi: 10.1016/j.jaci.2014.05.041 25062783

[34] Alsahali S. Awareness, views, perceptions, and beliefs of pharmacy interns regarding digital health in Saudi Arabia: cross-sectional study. JMIR Medical Education. 2021;7(3):e31149. doi: 10.2196/31149 34338649 PMC8449296

[35] Thapa S, Nielsen JB, Aldahmash AM, Qadri FR, Leppin A. Willingness to Use Digital Health Tools in Patient Care Among Health Care Professionals and Students at a University Hospital in Saudi Arabia: Quantitative Cross-sectional Survey. JMIR Med Educ. 2021;7(1):e18590. Epub 20210219. doi: 10.2196/18590 ; PubMed Central PMCID: PMC8081256. 33605896 PMC8081256

