# Correction: Knowledge and perception of mHealth medication adherence applications among pharmacists and pharmacy students in Jazan, Kingdom of Saudi Arabia

**DOI:** 10.1371/journal.pone.0332544

**Published:** 2025-09-11

**Authors:** Wajiha Rehman, Hemalatha Thanganadar, Sumaira Idrees, Asim Mehmood, Fahad Khan Azeez, Hanan Abdullah Almaimani, Pushp Lata Rajpoot, Mohammed Mustapha

There are errors in the Funding statement. The correct Funding statement is as follows: The authors received no specific funding for this work.
